# The patient journey of patients with Fabry disease, Gaucher disease and Mucopolysaccharidosis type II: A German-wide telephone survey

**DOI:** 10.1371/journal.pone.0244279

**Published:** 2020-12-31

**Authors:** Eugen Mengel, Jens Gaedeke, Holger Gothe, Simon Krupka, Anja Lachmann, Jörg Reinke, Christoph Ohlmeier

**Affiliations:** 1 SphinCS GmbH – Clinical Science for LSD, Hochheim, Germany; 2 Universitätsmedizin Mainz, Zentrum für Kinder- und Jugendmedizin, Villa Metabolica, Mainz, Germany; 3 Charité – Universitätsmedizin Berlin, Klinik für Nephrologie und Intensivmedizin, Berlin, Germany; 4 IGES Institut GmbH, Department Health Services Research, Berlin, Germany; 5 Chair for Health Sciences / Public Health, Medical Faculty “Carl Gustav Carus”, Technical University Dresden, Dresden, Germany; 6 Department of Public Health, Institute of Public Health, Medical Decision Making and Health Technology Assessment, Health Services Research and Health Technology Assessment, UMIT – University for Health Sciences, Medical Informatics and Technology, Tyrol, Austria; 7 Shire Deutschland GmbH, Berlin, Germany; 8 Medizinische Zentrum für Erwachsene mit Behinderung (MZEB) der Kreuznacher Diakonie, Bad Kreuznach, Germany; Univeristy of Tennessee, UNITED STATES

## Abstract

**Background:**

Lysosomal Storage Diseases (LSD) are rare and multisytemic diseases which are caused by lysosomal enzyme deficiencies leading into accumulation of waste products due to an interruption in the decomposition process. Due to the low prevalence and therefore limited disease awareness as well as the fact that LSD patients present with unspecific symptoms the final diagnosis is often made after a long delay. The aim of this German-wide survey was to characterize the period between onset of symptoms and final diagnosis regarding e.g. self-perceived health, symptom burden and false diagnoses for patients with selected LSDs (Fabry disease (FD), Gaucher disease (GD) and Mucopolysaccharidosis type II (MPS II).

**Methods:**

The study was conducted as a telephone based cross-sectional survey. All patients living in Germany with a confirmed diagnosis of FD, GD or MPS II were eligible to participate. The questionnaire was provided in advance in order to enable the participants to prepare for the interview. Only descriptive analyses were carried out. Single analyses were not carried out for all three patient groups due low case numbers.

**Results:**

Of the overall population, 39 patients have been diagnosed with FD, 19 with GD and 11 with MPS II with the majority of patients being index patients. The majority of FD patients reported their current health status as “satisfactory” or better (79.5%). Self-perceived health status was observed to be at least stable or improving for the majority of FD patients compared to the year prior to diagnosis. The most frequently reported symptoms for patients with FD were paraesthesias (51.3%), whereas patients with GD reported a tendency for bleeding, blue spots or coagulation disorder (63.2%) as well as hepatomegaly and/or splenomegaly (63.2%) as the most commonly appearing symptoms. The number of patients reporting misdiagnoses was n = 5 (13.5%) for patients with FD and n = 5 (27.8%) for patients with GD. The median duration of the diagnostic delay was 21.0 years for FD, 20.0 years for GD and 2.0 years for MPS II.

**Conclusions:**

This study showed that self-perceived status of health for patients might improve once the final correct diagnoses has been made and specific treatment was available. Furthermore, it was observed that diagnostic delay is still high in Germany for a relevant proportion of affected patients. Further challenges in the future will still be to increase awareness for these diseases across the entire healthcare sector to minimize the diagnostic delay.

## Introduction

Orphan or rare diseases are diseases with a particularly low prevalence of less than 5 out of 10,000 people. In Germany, the number of patients with a rare disease is estimated at four million [1]. Although rare diseases constitute a heterogeneous group of mostly complex disease patterns, they have in common that they can be life threatening, chronically debilitating and hereditary. In this study, we focused on Fabry disease, Gaucher disease and Hunter disease (Mucopolysaccharidosis type II; MPS II), which belong to the group of Lysosomal Storage Diseases (LSD). LSDs are caused by lysosomal enzyme deficiencies leading into accumulation of waste products due to an interruption in the decomposition process. LSDs are multisystemic diseases that can affect different organs. The diagnosis is therefore usually based on a combination of disease-related symptoms [2,3] which are confirmed by biochemical and genetic tests, which are in general not known by non-specialist physicians.

The prevalence of LSDs is estimated at 1 in 8,000 live births [4]. However, clinical heterogeneity complicates collecting reliable epidemiological data [5]. Considering the specific prevalence of the three LSDs focused in this study, Fabry disease is estimated to affect 1 to 5 per 10,000 persons [6], MPS II has an estimated prevalence of 1 in 162,000 live births in male individuals [7,8] and Gaucher disease affects approximately 1 to 9 in 100,000 people [9,10].

Due to unspecific symptoms, a relatively low prevalence and therefore limited disease awareness, there often is a comparatively long delay between the onset of the first symptoms and final diagnosis [3,11]. The period between first occurrence of symptoms and a reliable diagnosis is called the diagnostic delay. During the prolonged diagnostic phase individual symptoms are often attributed to other, more common diseases, which lead to incorrect diagnoses and possibly mistreatment [12]. For instance, in their differential diagnosis of patients presenting with the classic clinical features of Gaucher disease (anaemia, thrombocytopenia, hepatosplenomegaly and bone pain) physicians are more likely to consider leukaemia, lymphoma or multiple myeloma instead [3]. Misdiagnosis is common also in Fabry disease; rheumatological disease / rheumatic fever, arthritis and neuropsychological disorders are the leading previous misdiagnoses [13]. For patients, the phase of diagnostic delay may have an influence on the mood and psychological state. In a study with n = 462 Australian children living with rare diseases Zurynski et al. (2017) [14] found that consequences of delayed diagnosis include anxiety, loss of reproductive confidence because of an ill-defined genetic risk, frustration and stress (54%), disease progression (37%), delays in treatment (25%) and inappropriate treatments (10%).

During the past years, the phenomenon of diagnostic delay was subject to increased attention in research on rare diseases. The EurodisCare studies for example already addressed the issue explicitly, however, without considering LSD. Therefore, the aim of the present study is to characterize the time between the first symptoms and a final LSD diagnosis for patients within the German healthcare context. This publication is part of an overall work on rare diseases called “VISIBL Patient Journey”.

## Materials and methods

### Study design and patients

The study was conducted as a telephone-based cross-sectional survey. All patients with a confirmed diagnosis of Fabry, Gaucher disease type 1 or MPS II were potentially eligible to participate in the study. Diagnosis was confirmed based on self-reports of interviewed patients. No further inclusion or exclusion criteria were applied. The study was carried out from July 2017 to April 2018. The ethics committee of the Rhineland-Palatinate Chamber of Physicians approved the study conduct.

### Survey

The survey items (Table 1) were developed similarly to an already published European-wide study on the delayed diagnosis of rare diseases [15]. For additional items (e.g. subjective health status), validated scales were used [16].

**Table 1:** Variable definitions and categorizations in the survey.

Variable definition	Categorization
Rare disease in question	nominally scaled
Patient charakteristics	
Interviewpartner (Pat. selbst oder Angehöriger)	nominally scaled
Date of birth and age	nominally scaled
Beruflicher Status	nominally scaled
Höchster Schulabschluss	nominally scaled
Weitere betroffene Person in der Familie	nominally scaled
Aktueller selbst eingeschätzter Gesundheitszustand	ordinally scaled
Aktuelle selbsteingeschätzte Lebenszufriedenheit	ordinally scaled
Onset of first symptoms	
Age	metric
Date	nominally scaled
Frequency	metric
Physician contacts between symptom onset and final diagnosis	
Medical disciplin (type; number, frequency)	nominally scaled; metric
Hospitalizations between symptom onset and final diagnosis	
Hospital admission (number, frequency)	metric
Rescue Centre / Emergency Room (number, frequency)	metric
Diagnostic procedures due to symptomatic complaints	nominally scaled
Misdiagnoses between symptom onset and final diagnosis	
Number, frequency	metric
Medical disciplin through which the diagnosis was established	nominally scaled
Initiation of therapeutic measures due to misdiagnosis	
Drug prescrition (type; number, frequency)	nominally scaled; metric
Surgery (type; number, frequency)	nominally scaled; metric
Psychological therapy (type; number, frequency)	nominally scaled; metric
Other therapeutic measures (type; number, frequency)	nominally scaled; metric
First suspicion of a rare disease	
Age	metric
Date	nominally scaled
Person / institution that expressed the suspicion	nominally scaled
Final diagnosis	
Age	metric
Date	nominally scaled
Person / institution that expressed the suspicion	nominally scaled
State of health and life satisfaction before diagnosis	ordinally scaled
Communication of the diagnosis	
Person / institution that communicated the diagnosis	nominally scaled
Form of the communication	nominally scaled
Satisfaction with the way the diagnosis was communicated	ordinally scaled

Both sources were used to develop an inventory of questions suitable for the study. The questionnaire has not been validated against medical records, instruments used in other studies or other documentations. To ensure feasibility of the questionnaire a two-stage pretest was conducted. The first step focused on the feasibility of the telephone interview in the designed form, the comprehensibility of the items, the formal answerability of the questions as well as the time required. In the second step, a revised interview guideline based on the findings of the first step was tested and approved as a result. To avoid further limitation of the patient population eligible for this study, the pretest was performed on persons who were not affected by one of the three considered LSDs.

### Study procedure

Study participants were recruited via three different approaches. Patients either were approached by treating physicians or contact was established via patient advocacy groups. In addition, home therapy providers introduced the study to patients and informed on the opportunity to participate. In case of interest, eligible patients were provided with information on the study encompassing informative letters, a declaration of consent including information regarding the handling of data to be collected as well as appointment forms and the questionnaire. Patients willing to participate in the study contacted the IGES Institute with the signed declaration of written informed consent and suggested dates for a telephone call. The questionnaire was provided in advance in order to enable the participants to prepare for the interview (e.g. sorting documents, contacting family members). A trained interviewer carried out all telephone interviews. Survey items included questions regarding sociodemographic information, self-perceived health, clinical symptoms, utilization of healthcare services, and initial suspicion of the presence of a rare disease as well as the diagnostic process. Interviews with patients who, due to their state of health, were not able to conduct the interview themselves, were conducted with the parents of the patients concerned after obtaining their consent. Nearly all information collected related to the time before the diagnosis. Each interview lasted an average of 30 minutes.

### Statistical analysis

Descriptive analysis of the data obtained from the study population was performed. Proportional values as well as measures of central tendency and corresponding measures of dispersion were calculated. Analyses were carried out separately for each patient population.

## Results

### Characteristics

According to Table 2, n = 69 LSD patients have been included in the analysis. Of the overall population, n = 39 patients have been diagnosed with Fabry, n = 19 with Gaucher disease and n = 11 with MPS II. Mean age of patients was 50.8 years (SD: 14.1) for patients with Fabry, 53.9 years (SD: 20.8) for patients with Gaucher disease and 22.3 years (SD: 16.8) for patients with MPS II. A little more than half of the Fabry and Gaucher patients were female. Due to characteristics of MPS II, all patients in this group were male. Within all three groups, the majority of persons were index patients, which means they were the first person in their family diagnosed with the disease. Regarding the occupational status, most of the Fabry and Gaucher patients were either employed (43.6% Fabry; 31.6% Gaucher) or pensioned (25.6% Fabry; 42.1% Gaucher), whereas most of the patients with MPS II were still in school or university (45%) or mentioned “others” as their occupational status (27%).

**Table 2:** Characteristics of patients with specifc lysosomal storage diseases.

	Fabry disease (n = 39)		Gaucher disease (n = 19)		MPS II (n = 11)	
	n	%	n	%	n	%
**Age**						
0–19 years	2	5.1%	2	10.5%	6	54.5%
20–39 years	2	5.1%	2	10.5%	3	27.3%
40–59 years	25	64.1%	6	31.6%	2	18.2%
60–79 years	10	25.6%	7	36.8%	0	0.0%
80+ years	0	0.0%	2	10.5%	0	0.0%
All	39	100.0%	19	100.0%	11	100.0%
Mean age (mean, SD)	50.8	14.1	53.9	20.8	22.3	16.8
**Sex**						
Women	21	53.8%	11	57.9%	0	0.0%
Men	18	46.2%	8	42.1%	11	100.0%
All	39	100.0%	19	100.0%	11	100.0%
**Index patient** ^a^*						
Yes	21	56.8%	15	78.9%	8	72.7%
No	16	43.2%	4	21.1%	3	27.3%
All	37	100.0%	19	100.0%	11	100.0%
**Current occupation**						
Pupil / university student	2	5.1%	2	10.5%	5	45.5%
Apprentice	0	0.0%	0	0.0%	0	0.0%
Employed	17	43.6%	6	31.6%	0	0.0%
Pensioner	10	25.6%	8	42.1%	2	18.2%
Job-seeking	1	2.6%	0	0.0%	0	0.0%
Incapacitated	7	17.9%	2	10.5%	1	9.1%
Others	2	5.1%	1	5.3%	3	27.3%
All	39	100.0%	19	100.0%	11	100.0%

SD: Standard deviation; MPS: Mucopolysaccharidosis.

^a^ First person in the family to be affected by the rare disease.

*n = 2 with missing information (Fabry disease).

### Health status

In the study, the patients were asked about their self-perceived state of health. The survey questions addressed both, the current state of health (“How would you describe your current state of health?”) as well as the state of health one year before diagnosis ("If you think of the year before the final diagnosis, how would you describe your state of health during that period?”). Due to low case numbers within the Gaucher and MPS II group, the association of current and past health status was analyzed for Fabry patients only (Table 3). The contingency table shows the distribution of the current health status as a function of the health status before the diagnosis. Constellations in which patients classify their current state of health at least as well as in the year before the final diagnosis are colored in grey. The table reflects that the majority of patients with Fabry disease did not feel any deterioration in their general health status despite suffering from a progressive disease. Especially the group of patients who described their health status as “poor” or “less well” seemed to be much smaller once they got a final diagnosis.

**Table 3:** Association between current state of health and the state of health one year before diagnosis in patients with Fabry disease (n = 39).

		State of health one year before diagnosis (self-rated)											
		very good		good		satisfactory		less well		poor		All	
		n	%	n	%	n	%	n	%	n	%	n	%
Current state of health (self-rated)	very good	1	50.0%	2	12.5%	0	0.0%	0	0.0%	1	9.1%	4	100%
	good	1	50.0%	7	43.8%	1	20.0%	1	20.0%	2	18.2%	12	100%
	satisfactory	0	0.0%	5	31.3%	3	60.0%	3	60.0%	4	36.4%	15	100%
	less well	0	0.0%	1	6.3%	0	0.0%	1	20.0%	3	27.3%	5	100%
	poor	0	0.0%	1	6.3%	1	20.0%	0	0.0%	1	9.1%	3	100%
	All	2	100%	16	100%	5	100%	5	100%	11	100%	39	100%

Note: Underlining indicates such constellations in which patients currently classify their state of health at least as well as in the year before the final diagnosis.

### Symptoms

Participants were asked to state which symptoms they had suffered from before they got their final diagnosis. Furthermore, the frequency at which these symptoms occurred as well as the age of their first manifestation were assessed. Based on expert knowledge, a categorization of the described symptoms was carried out in order to ensure a uniform designation.

**Fig 1:**
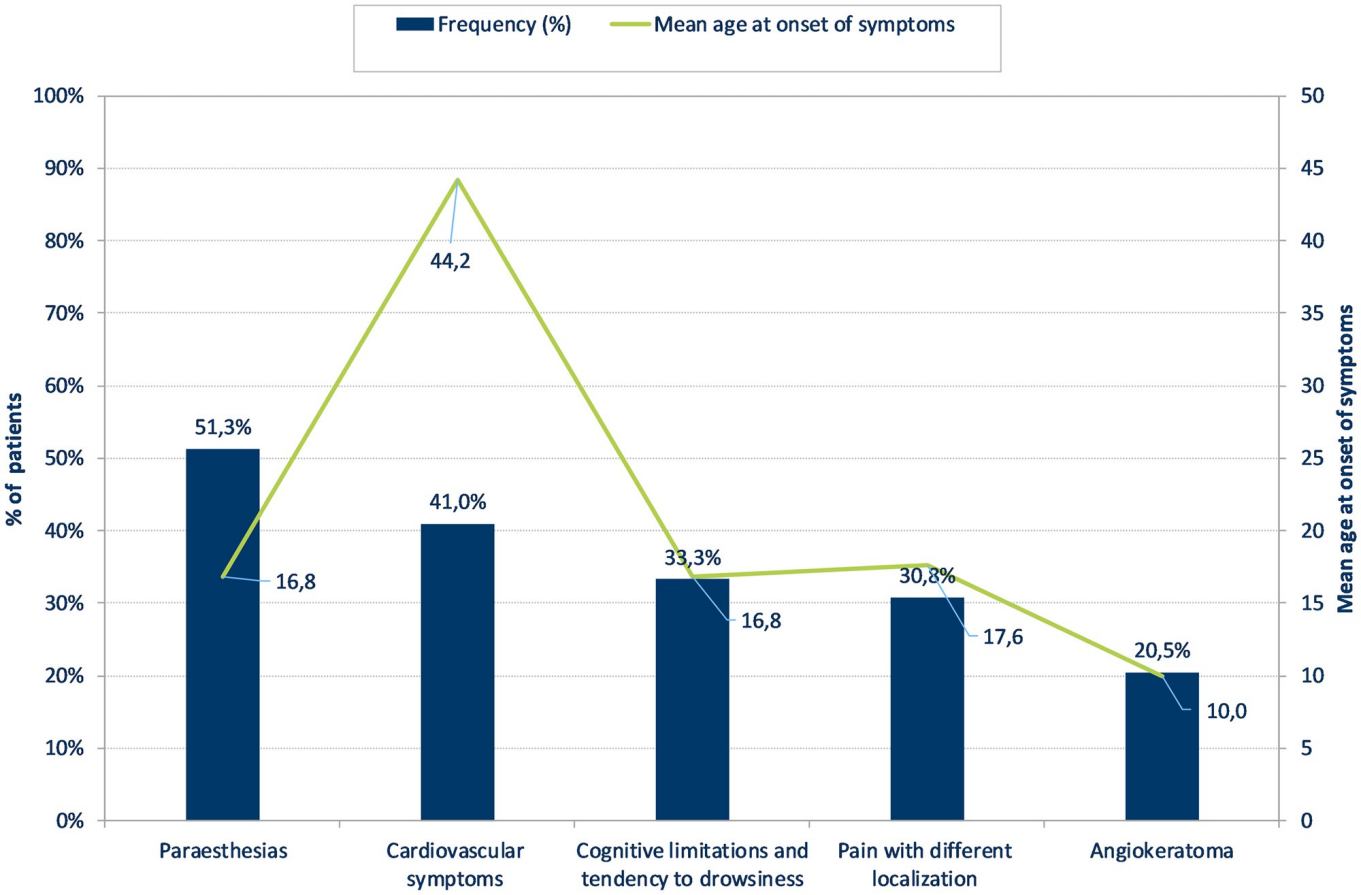
Prevalence and age at occurrence of the five most frequently reported symptoms in patients with Fabry disease (n = 39).

**Fig 2:**
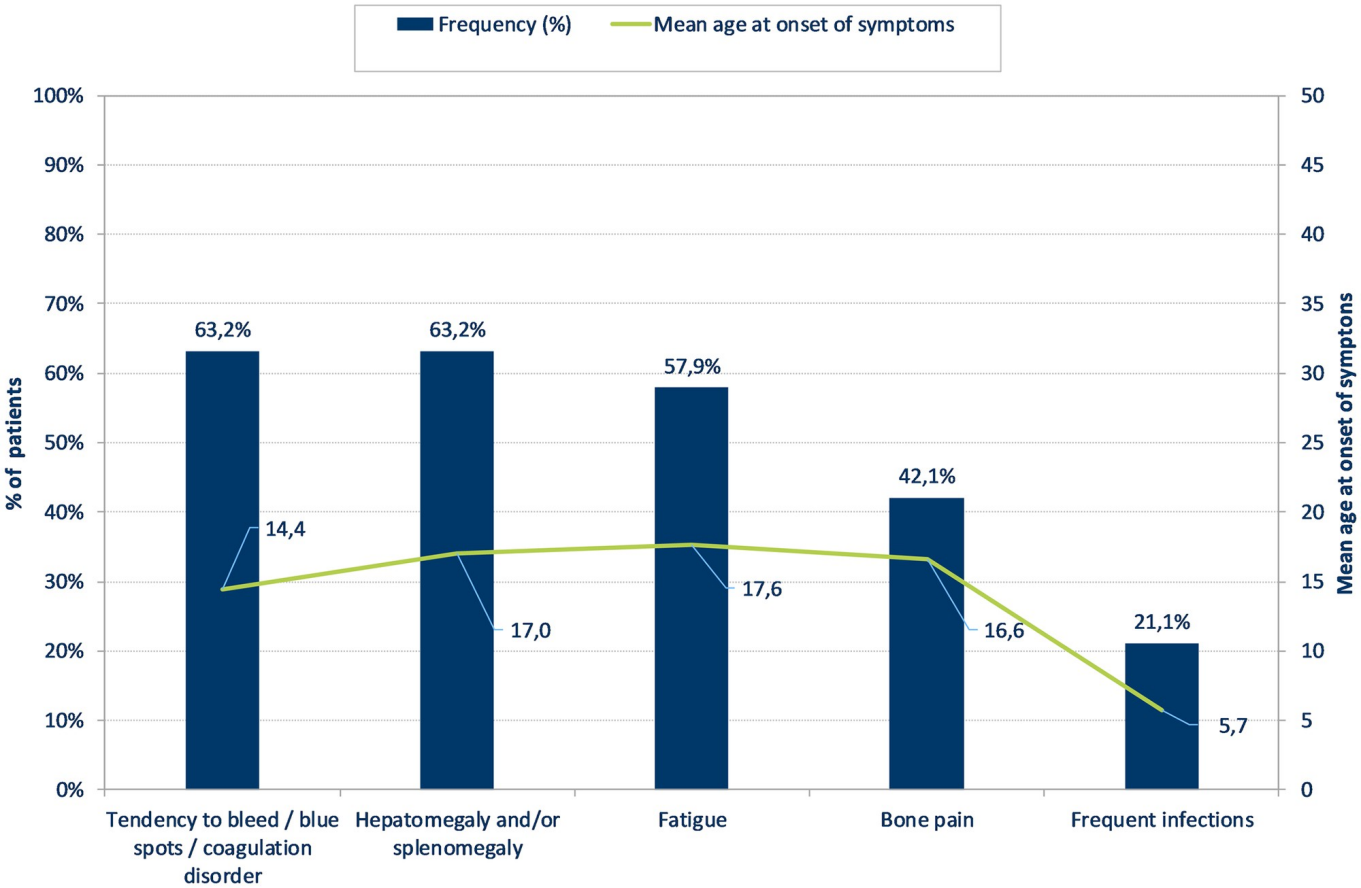
Prevalence and age at occurrence of the five most frequently reported symptoms in patients with Gaucher disease (n = 19).

Figs 1 and 2 show the five most frequently reported symptoms for patients with Fabry and Gaucher disease, respectively. The most frequently reported symptoms for patients with Fabry disease were paraesthesias (51.3%), whereas patients with Gaucher disease reported a tendency for bleeding, blue spots or coagulation disorder (63.2%) as well as hepatomegaly and/or splenomegaly (63.2%) as the most commonly appearing symptoms. Mean age at onset of the symptoms showed a range between 10.0–44.2 years for Fabry, and 5.7–17.6 years for Gaucher patients.

Due to the relatively small number of participants, results for MPS II are not reported.

### Misdiagnoses

As stated earlier, diagnostics in rare diseases can be challenging and bears a risk of misdiagnosis possibly accompanied by mistreatment. Therefore, study participants were asked whether they were initially diagnosed differently due to alternative interpretation of the diverse disease symptoms. Within this study, the existence of misdiagnosis was assumed if presence of another disease was presumed, at least for a short time.

In contrast, a suspected diagnosis that has been made during the patient examination, but not confirmed, was not considered as a false diagnosis. The number of patients reporting misdiagnoses was n = 5 (13.5%) for patients with Fabry disease and n = 5 (27.8%) for patients with Gaucher disease (Table 4). For Fabry disease, reported misdiagnoses were gastritis, mental disorder, rheumatism, chronic polyarthritis and multiple sclerosis, whereas Gaucher patients reported rheumatism, malignant blood disease/leukemia, anemia, benign blood disease and tonsillitis as misdiagnoses.

**Table 4:** Misdiagnoses in patients with Fabry disease (n = 39) and Gaucher disease (n = 19).

	Fabry disease (n = 39)		Gaucher disease (n = 19)	
	n	%	n	%
**Patients reporting misdiagnoses**				
Yes	5	13.5%	5	27.8%
No	32	86.5%	13	72.2%
All*	37	100.0%	18	100.0%

*n = 2 with missing information for Fabry disease; n = 1 with missing information for Gaucher disease.

Additionally, the interviewed patients provided information on the phase in which the Fabry or Gaucher disease was suspected for the first time. The mean age at first suspicion was 39.6 years (SD: 15.1) for Fabry patients and 26.7 years (SD: 17.2) for Gaucher patients. The first suspicion of Fabry and Gaucher disease was most frequently made in a hospital (43.6% and 52.6% respectively), followed by specialists (25.6% and 21.1% respectively) and family members (20.5% and 10.5% respectively). First suspicion by a general practitioner was reported for a proportion of 5.1% of the Fabry patients and 10.5% of the Gaucher patients. The final diagnoses was made at a mean age of 41.1 years (SD: 14.4) for Fabry disease and 28.2 years (SD: 17.9) for Gaucher disease and was most frequently made shortly after the first suspicion had been raised. Both diseases were most frequently diagnosed in a hospital (38.5% and 63.2% respectively). Fabry disease diagnosis was often made in specialized centers (35.9%) (Table 5).

**Table 5:** Person group/institution with final diagnosis in patients with Fabry disease and Gaucher disease.

	Fabry disease (n = 39)		Gaucher disease (n = 19)	
	n	%	n	%
**Person group / institution**				
General practitioner	2	5.1%	1	5.3%
Specialist	7	17.9%	2	10.5%
Specialized center	14	35.9%	4	21.1%
Hospital	15	38.5%	12	63.2%
Other health professions	0	0.0%	0	0.0%
Others	1	2.6%	0	0.0%
All	39	100.0%	19	100.0%
Mean age at final diagnosis (mean, SD)	41.1	14.4	28.2	17.9

SD: Standard deviation.

### Diagnostic delay

From information on the onset of initial symptoms and the date of final diagnosis, we calculated the diagnostic delay for each patient group. The median duration of the diagnostic delay was 21.0 years (IQR: 21.0) for Fabry disease, 20.0 years (IQR: 32.0) for Gaucher disease and 2.0 years (IQR: 5.0) for MPS II. Considering the diagnostic delay for specific age groups, it is noticeable that patients in older age groups usually had a higher diagnostic delay. Furthermore, the diagnostic delay in index patients was longer than in non-index patients. For patients with Fabry disease, where the mean duration of the diagnostic delay was 26.0 years for index and 18.0 years for non-index patients, this difference was most visible (Table 6).

**Table 6:** Diagnostic delay of patients with specifc lysosomal storage diseases.

	Fabry disease (n = 39)					Gaucher disease (n = 19)					MPS II (n = 11)				
	n	Mean	SD	Median	IQR	n	Mean	SD	Median	IQR	n	Mean	SD	Median	IQR
**Age at onset of first symptoms**															
Women	21	23.3	21.3	11.0	38.5	11	13.5	12.7	7.0	17.0	0	/	/	/	/
Men	18	12.8	12.9	7.0	13.5	8	11.3	9.7	6.0	17.3	11	1.2	1.2	1.0	2.0
All	39	18.4	18.7	10.0	28.0	19	12.6	11.6	7.0	17.0	11	1.2	1.2	1.0	2.0
**Age at final diagnosis**															
Women	21	40.6	15.8	40.0	18.5	11	25.4	14.3	27.0	33.0	0	0	0	0	0
Men*	18	41.7	12.2	39.5	21.5	8	32.0	21.3	38.0	46.8	10	28.2	17.9	3.0	4.75
All	39	41.1	14.4	40.0	19.0	19	28.2	17.9	28.0	36.0	10	28.2	17.9	3.0	4.75
**Diagnostic delay (in years)**															
Overall	39	26.1	22.4	21.0	21.0	19	20.0	17.3	20.0	32.0	10	5.3	9.5	2.0	5.0
By age group **															
0–19 years	1	1.0	0.0	1.0	/	2	2.5	2.5	2.5	/	5	1.8	1.6	2.0	3.5
20–39 years	2	20.0	6.0	20.0	/	2	1.5	0.5	1.5	/	3	1.0	0.8	1.0	2.0
40–59 years	25	22.4	14.4	21.0	21.0	6	19.5	15.2	19.5	2.3	2	20.5	12.5	20.5	/
60–79 years	9	24.2	16.9	24.0	29.5	7	25.7	16.7	26.0	5.0	0	/	/	/	/
≥80 years	0	/	/	/	/	2	37.5	9.5	37.5	/	0	/	/	/	/
All	37					19					10				
By index patient***															
Yes	23	24.5	14.8	26.0	19.0	15	20.0	16.6	20.0	28.0	7	6.9	10.9	2.0	8.0
No	16	28.4	29.9	18.0	27.3	4	20.0	19.8	18.0	41.5	3	1.7	1.7	1.0	4.0
All	39					19					10				

^"/"^: No values available due to low case numbers.

SD: Standard deviation; IQR: Interquartile range.

*n = 1 with missing information (MPS II).

** n = 2 with missing information (Fabry disease), n = 1 with missing information (MPS II).

*** n = 1 with missing information (MPS II).

## Discussion

In this study, we interviewed 69 patients with LSD in order to trace their journey through their phase of diagnostic delay. Within the overall group of LSD patients, we considered 39 patients with Fabry disease, 19 patients with Gaucher disease and 11 patients with MPS II.

Comparing the study sample of the present study to those described in other publications such as the Fabry Outcome Survey registry (FOS registry), the Gaucher Outcome Survey registry (GOS registry) and the Hunter Outcome Survey registry (HOS registry) [13,17,18], the mean age of patients in this study was higher which is likely due to differing data sources and a selective inclusion on index patients in our study. Registries usually report the age for inclusion into the registry which occurs either at the time of diagnosis or at treatment initiation. In our study patients reported their current age. Thus, differences were to be expected. Additionally, the comparatively high mean age observed in this study may be an indication for a higher willigness to participate of index patients, who are likely to be older compared to non-index patients and generally should habe a high interest in the research question of this study. When comparing the proportion of participating index patients from our study with those reported from registries, it becomes apparent that the proportion of index patients in our study was relatively high [13,17,18] which primarily leads to a comparatively high diagnostic delay observed in this study (details discussed below).

The length of the diagnostic delay, especially for Fabry patients, seems noticeably higher in our study than in the FOS registry [11]. While the median diagnostic delay in this study was 21.0 years, patients included in the FOS registry were waiting 10.5 to 14.0 years for their final diagnosis [11]. Metha et al. (2004) for example reported a diagnostic delay of 13.7 years for male and 16.3 years for female index patients with Gaucher disease, which is shorter than in our study. Furthermore, an age-dependent gradient was shown in this study, with persons at higher ages having a longer diagnostic delay. As discussed before, this difference is mainly due to a more frequent inclusion of index patients in our study. This is underlined by the fact that subgroup analyses revealed a longer diagnostic delay for the index- versus non-index patients for all three populations. Nevertheless, the observed diagnostic delay for index patients still was comparatively high and is therefore reality for a relevant proportion of patients.

Compared to previous decades, the diagnosis of rare diseases is now made much earlier due to increased awareness, which has been exemplarily shown for hereditary angioedema [19]. This fact is also supported by the fact that in our study a shorter diagnostic delay was observed in younger patients compared to older patients. At the same time, it is also a reality that even young patients in some cases still have to wait a long time for a diagnosis and an effective treatment. Therefore, an increase in awareness should still be aimed for.

The present study also assessed the self-perceived state of health of Fabry patients before and after the final diagnosis. Interestingly, with regard to the change before and after the final diagnosis, it becomes apparent that the number of patients rating their health before the final diagnoses as satisfactory or better increases, once the final diagnoses has been made. The elimination of uncertainty, the fact that patients feel taken seriously and the availability of effective treatment options is likely to contribute to the increase of “satisfactory” or even better self-perceived health status after final diagnosis. This was also a frequently expressed aspect in the survey situation. Furthermore, the majority of patients with Fabry disease did not feel any deterioration in their health, despite having a progressive disease.

Receiving a misdiagnosis is serious for rare disease patients because it prevents them from receiving effective treatment, may result in ineffective or unnecessary therapy, and leads to the fact that family members cannot be adequately informed. The proportion of patients receiving misdiagnoses was 13.5% for Fabry and 27.8% for Gaucher patients. Findings on the frequency of misdiagnoses are available in the research context especially for Fabry disease and corresponding results are higher than findings from the present study [13]. The discrepancy might be attributable to the definition of a false diagnosis. In the present survey, a conservative definition of false diagnoses was used. If a less conservative definition of misdiagnosis was applied in other studies, this may have led to an underestimation of the frequency of misdiagnoses in the present study.

The results on the institution that first suspected the correct disease/diagnosis illustrate that the majority of patients receive their diagnosis within hospitals and specialized centers. Hospital based physicians (43.6% Fabry; 52.6% Gaucher) or specialists (25.6% Fabry; 21.1% for Gaucher) made the first suspicion for most of the Fabry and Gaucher patients. First suspicion raised by a general practitioner was reported for a smaller proportion of patients (5.1% Fabry; 10.5% Gaucher). Interestingly in a relatively high number of patients with Fabry the disease was first susptected by family members (20.5%), which might reflect answers of non-index patients with a family history of the disease. The final diagnosis then again was primarily made in hospitals or in specialized treatment centers. However, survey participants might have interpreted the concept of a specialized center differently than intended. Respondents assumed treatment in a specialized center also if they have been treated in a special department of a hospital. Therefore, the proportion of patients that were treated in a specialized center might be overestimated by the results of this study and, vice versa, the treatment in hospitals might be underestimated.

### Strengths and limitations

The limitations of the present study mainly include the selective participation of participants and furthermore, restrictions associated with collecting information via telephone. The limitation mentioned first is mainly described by a higher recruitment of index patients, older patients and patients with a special interest in the research question into the study sample. The recruitment of patients via treating physicians may have caused a bias as patients that were included in the survey may not necessarily be representative of the overall patient population. In addition, of those patients who were contacted for involvement, the ones that did participate may have had different experiences compared to those who declined participation. Therefore, the results of this study must be interpreted against the background of bounded validity of the collected data. A fully representative sample of Fabry, Gaucher and MPS II patients cannot be presumed. This particularly concerns the results on the length of the diagnostic delay but could also affect results for example on the reported proportion of misdiagnoses.

Additionally, an interpretation of the results must consider that the information was collected retrospectively as part of a telephone interview based survey. It is therefore likely that due to a recall bias, information or recollections of past events have not been reported or reproduced adequately. In particular, reported symptoms prior to final diagnosis and the utilization of the health care system prior to correct diagnosis are likely to be underestimated.

## Conclusion

This study aimed to characterize the time between first symptoms and a final diagnosis, called the diagnostic delay, for patients with Fabry disease, Gaucher disease and MPS II within the German healthcare context. Although the duration of the diagnostic delay, which mainly occurs due to the largely unspecific symptoms, eported in this study could be influenced by the sample composition, the study results still show that there is still a long delay in diagnosing patients with rare diseases even in younger age groups. Considering that the self-perceived state of health might improve after receiving the diagnosis due to e.g. the reductions of uncertainty, further efforts should be made in the future to reduce the diagnostic delay and therefore allowing these patients timely access to effective treatment options which can lead to improved patient outcomes. Therefore, further challenges in the future will still be to increase awareness for these diseases across the entire healthcare sector to minimize the diagnostic delay.

## Supporting information

S1 File Survey questionnaire in German. (PDF)

S2 File Survey questionnaire in English. (PDF)

## References

[1] Bundesgesundheitsministerium für Gesundheit. Seltene Erkrankungen 2019 [updated 27.05.201916.08.2019]. https://www.bundesgesundheitsministerium.de/themen/praevention/gesundheitsgefahren/seltene-erkrankungen.html#c3521.

[2] Wenger DA, Luzi P, Rafi MA. Lysosomal storage diseases: heterogeneous group of disorders. BioImpacts. 2013;3(4): 145–147. 10.5681/bi.2013.029 24455477 PMC3892733

[3] Mehta A, Belmatoug N, Bembi B, Deegan P, Elstein D, Goker-Alpan O, et al Exploring the patient journey to diagnosis of Gaucher disease from the perspective of 212 patients with Gaucher disease and 16 Gaucher expert physicians. Mol Genet Metab. 2017;122(3): 122–129. 10.1016/j.ymgme.2017.08.002 28847676

[4] Schultz ML, Tecedor L, Chang M, Davidson BL. Clarifying lysosomal storage diseases. Trends Neurosci. 2011;34(8): 401–410. 10.1016/j.tins.2011.05.006 21723623 PMC3153126

[5] Fuller M, Meikle PJ, Hopwood JJ. Epidemiology of lysosomal storage diseases: an overview. Mehta A, Beck M, Sunder-Plassmann G, editors: Oxford PharmaGenesis; 2006. 21290699

[6] OrphaNet. Fabry Disease 2012. https://www.orpha.net/consor/cgi-bin/Disease_Search.php?lng=DE&data_id=94.

[7] OrphaNet. MPS II 2007. https://www.orpha.net/consor/cgi-bin/Disease_Search.php?lng=EN&data_id=131.

[8] Kampmann C, Beck M, Morin I, Loehr JP. Prevalence and Characterization of Cardiac Involvement in Hunter Syndrome. J Pediatr. 2011;159(2): 327–331. 10.1016/j.jpeds.2011.01.054 21529823

[9] OrphaNet. Gaucher Disease 2006. https://www.orpha.net/consor/cgi-bin/Disease_Search.php?lng=DE&data_id=644.

[10] Beck M, vom Dahl S, Mengel E, Niederau C, Poll L, Arndt R. Leitlinie zur Diagnostik und Therapie des M Gaucher. 20074. https://www.orpha.net/consor/cgi-bin/Disease_Search.php?lng=EN&data_id=131.

[11] Resin R, Perrin A, Garcia-Pavia P. Time delays in the diagnosis and treatment of Fabry disease. Int J Clin Prac. 2017;71(1). 10.1111/ijcp.12914 28097762

[12] Frank M, Eidt-Koch D, Aufmann I, Reimann A, Wagner TO, von der Schulenburg GJM. Measures to improve the healthsituation of patients with rare diseases in Germany: A comparison with the National Action Plan. Bundesgesundheitsblatt. 2014;57(10): 1216–1223. 10.1007/s00103-014-2040-2 25209683

[13] Mehta A, Ricci R, Widmer U, Dehout F, Garcia de Lorenzo A, Kampmann C, et al Fabry disease defined: baseline clinical manifestations of 366 patients in the Fabry Outcome Survey. Eur J Clin Invest. 2004;34(3): 236–242. 10.1111/j.1365-2362.2004.01309.x 15025684

[14] Zurynski Y, Deverell M, Dalkeith T, Johnson S, Christodoulou J, Leonard H, et al Australian children living with rare diseases: experiences of diagnosis and perceived consequences of diagnostic delays. Orphanet J Rare Dis. 2017;12(68). 10.1186/s13023-017-0622-4 28399928 PMC5387276

[15] Kole A, Faurisson F. The Voice of 12,000 Patients. Experiences and Expectations of Rare Disease Patients on Diagnosis and Care in Europ: Eurodis; 2009. p. 324.

[16] German Socio-Economic Panel (SOEP). TNS Infratest Sozialforschung. Erhebungsinstrumente des IAB-SOEP Migrationssamples 2016. 2193–5580. Berlin: 2016.

[17] Wraith JE, Beck M, Giugliani R, Clarke J, Martin R, Muenzer J. Initial report from the Hunter Outcome Survey. Genet Med. 2008;10(7): 508–516. 10.1097/gim.0b013e31817701e6 18580692

[18] Zimran A, Belmatoug N, Bembi B, Deegan P, Elstein D, Fernandez-Sasso D, et al Demographics and patient characteristics of 1209 patients with Gaucher disease: Descriptive analysis from the Gaucher Outcome Survey (GOS). Am J Hematol. 2018;93(2): 205–212. 10.1002/ajh.24957 29090476 PMC5814927

[19] Zanichelli A, Magerl M, Longhurst HJ, Aberer W, Caballero T, Bouillet L, et al Improvement in diagnostic delays over time in patients with hereditary angioedema: findings from the Icatibant Outcome Survey. Clin Transl Allergy. 2018;8:42 10.1186/s13601-018-0229-4 30338053 PMC6182796

