# Supplementary material for: The patient journey of patients with Fabry disease, Gaucher disease and Mucopolysaccharidosis type II: A German-wide telephone survey
Source: PLoS One. 2020 Dec 31;15(12):e0244279. doi: 10.1371/journal.pone.0244279 (PMC7775043; doi:10.1371/journal.pone.0244279)
Supplement: S1 File — (PDF) [file pone.0244279.s001.pdf]

Befragung im Rahmen der VISIBL-Studie zu seltenen Erkrankungen

Bitte beantworten Sie folgende Fragen und halten den Fragebogen für die telefonische Befragung bereit, damit wir zu diesem wichtigen Thema möglichst alle Informationen von Ihnen erhalten.

Bitte lesen Sie die Fragen sorgfältig durch. In den Fragen erhalten Sie zusätzliche Informationen darüber, ob z. B. mehrere Antwortmöglichkeiten angekreuzt werden können oder ob Sie einzelne Fragen überspringen können und nicht beantworten müssen. Alle Fragen richten sich an den Patienten selbst. Sollte also eine weitere Person an der Befragung beteiligt sein bzw. die Befragung übernehmen, achten Sie bitte darauf, dass alle Fragen aus der Perspektive des Patienten beantwortet werden.

Diesen Fragebogen bitte nicht mitsenden! Der Fragebogen dient lediglich zu Ihrer Vorbereitung auf die telefonische Befragung.

Vielen Dank für Ihre Mitarbeit!

Bei Fragen zur Durchführung der telefonischen Befragung wenden Sie sich bitte an:

Dr. Christoph Ohlmeier
IGES Institut GmbH
Friedrichstr. 180
10117 Berlin
Tel: 030 230809-0
Mail: christoph.ohlmeier@iges.com

Bei medizinischen Fragen in Zusammenhang mit dem Fragebogen wenden Sie sich je nach vorliegender seltener Erkrankung bitte an einen der folgenden Ärzte:

Hereditäres Angioödem
Prof. Dr. Markus Magerl
Charité Universitätsmedizin Berlin
Tel: 030 450 518 318
Mail: markus.magerl@charite.de

Morbus Fabry
Dr. Jens Gaedeke
Charité Universitätsmedizin Berlin
Tel: 030 450 614137
Mail: jens.gaedeke@charite.de

Morbus Gaucher
Dr. Eugen Mengel
Villa Metabolica / Universitätsmedizin Mainz
Tel: 06131 17 5754
Mail: karl-eugen.mengel@unimedizin-mainz.de

Morbus Hunter / Mukopolysaccharidose Typ II
Dr. Jörg Reinke
Villa Metabolica / Universitätsmedizin Mainz
Tel: 06131 17 5754
Mail: joerg.reinke@unimedizin-mainz.de

A Angaben zur Person

A.1 Mit wem wird die telefonische Befragung durchgeführt?

- ❑ Betroffene Person selbst
- ❑ Minderjährige betroffene Person (<18 Jahre) gemeinsam mit Erziehungsberechtigtem/-n
- ❑ Erziehungsberechtigte/-r in Vertretung der betroffenen Person
- ❑ Volljährige betroffene Person gemeinsam mit Bezugsperson oder rechtlichem Betreuer

A.2 Wann sind Sie geboren?

__ __/__ __ __ __
M M / J J J J

A.3 Ihr Geschlecht:

Männlich ❑ Weiblich ❑

A.4 Welchen Beruf üben Sie derzeit aus?

- ❑ Schüler/Student
- ❑ Auszubildender
- ❑ Berufstätig
- ❑ Rentner
- ❑ Arbeitsuchend
- ❑ Berufsunfähig
- ❑ Anderes *(bitte angeben)*:

____

A.5 Welches ist der höchste Bildungsabschluss von Ihnen?

- ❑ Kein Schulabschluss
- ❑ Haupt- oder Realschulabschluss bzw. gleichwertiger Schulabschluss
- ❑ Abitur
- ❑ Fachhochschulabschluss
- ❑ Universitätsabschluss
- ❑ Promotion
- ❑ Anderes *(bitte angeben)*:

____

A.6 **Welche Person war vorrangig bei Ihren Arztkontakten in Zusammenhang mit Ihrer seltenen Erkrankung involviert?**<br><br>❑ Elternteil<br>❑ Ehe- oder Lebenspartner<br>❑ Anderes Familienmitglied<br>❑ Freunde/Bekannte<br>❑ Ich habe dies vorrangig selbst organisiert<br>❑ Andere *(bitte angeben)*: ____________________	A.7 **Welches ist der höchste Bildungsabschluss von dieser Person?**<br><br>❑ Kein Schulabschluss<br>❑ Haupt- oder Realschulabschluss bzw. gleichwertiger Schulabschluss<br>❑ Abitur<br>❑ Fachhochschulabschluss<br>❑ Universitätsabschluss<br>❑ Promotion<br>❑ Anderes *(bitte angeben)*: ____________________

A.8 **Welche seltene Erkrankung liegt bei Ihnen vor?**

❑ Morbus Fabry
❑ Morbus Gaucher
❑ Morbus Hunter/Mukopolysaccharidose Typ II
❑ Hereditäres Angioödem

A.9 **Sind in Ihrer Familie weitere Personen von dieser seltenen Erkrankung betroffen?**

❑ Ja ❑ Nein

A.10 **Wie würden Sie gegenwärtig Ihren Gesundheitszustand beschreiben?**

❑ Sehr gut
❑ Gut
❑ Mittelmäßig
❑ Weniger gut
❑ Schlecht
❑ Keine Angabe

A.11 **Wie zufrieden sind sie gegenwärtig, alles in allem, mit Ihrem Leben?**

Bitte auf der Skala von 0 („ganz und gar unzufrieden“) bis 10 („ganz und gar zufrieden“) ankreuzen!

0	1	2	3	4	5	6	7	8	9	10
❑	❑	❑	❑	❑	❑	❑	❑	❑	❑	❑

B Zeit vom Auftreten erster Symptome bis zur finalen Diagnosestellung

B.1 Was waren die ersten Symptome der seltenen Erkrankung, die bei Ihnen auftraten?

**Symptom**	**Wann ist das Symptom das erste Mal aufgetreten?**	**Wie oft trat das Symptom vor der finalen Diagnosestellung auf?**			
		1- bis 5-mal	6- bis 20-mal	Mehr als 20-mal	Dauer-haftes Symp-tom
	Alter: ______ oder<br>Datum: ____/____ (Monat/Jahr)	❑	❑	❑	❑
	Alter: ______ oder<br>Datum: ____/____ (Monat/Jahr)	❑	❑	❑	❑
	Alter: ______ oder<br>Datum: ____/____ (Monat/Jahr)	❑	❑	❑	❑
	Alter: ______ oder<br>Datum: ____/____ (Monat/Jahr)	❑	❑	❑	❑
	Alter: ______ oder<br>Datum: ____/____ (Monat/Jahr)	❑	❑	❑	❑

B.2 Wie viele verschiedene Ärzte haben Sie zwischen dem Auftreten erster Symptome und der finalen Diagnosestellung aufgrund Ihrer Beschwerden aufgesucht?

❑ 1 bis 2 Ärzte

❑ 3 bis 5 Ärzte

❑ 6 bis 10 Ärzte

❑ 11 bis 20 Ärzte

❑ Mehr als 20 Ärzte

B.3 **Wie häufig haben Sie zwischen dem Auftreten erster Symptome und der finalen Diagnosestellung aufgrund Ihrer Beschwerden die einzelnen im Folgenden genannten Arztgruppen besucht?**

	0 mal	1- bis 2-mal	3- bis 5-mal	6- bis 10-mal	11- bis 20-mal	Mehr als 20-mal
Hausarzt	❑	❑	❑	❑	❑	❑
Neurologe	❑	❑	❑	❑	❑	❑
Kardiologe	❑	❑	❑	❑	❑	❑
Nephrologe	❑	❑	❑	❑	❑	❑
Hals-Nasen-Ohren-Arzt	❑	❑	❑	❑	❑	❑
Orthopäde	❑	❑	❑	❑	❑	❑
Hämatologe	❑	❑	❑	❑	❑	❑
Gynäkologe	❑	❑	❑	❑	❑	❑
Pädiater/Kinderarzt	❑	❑	❑	❑	❑	❑
Dermatologe/Hautarzt	❑	❑	❑	❑	❑	❑
Allergologe	❑	❑	❑	❑	❑	❑
Gastroenterologe/Hepatologe	❑	❑	❑	❑	❑	❑
Augenarzt	❑	❑	❑	❑	❑	❑
Rheumatologe	❑	❑	❑	❑	❑	❑
Psychologe/Psychiater	❑	❑	❑	❑	❑	❑
Andere	❑	❑	❑	❑	❑	❑

B.4 **Wie häufig waren Sie zwischen dem Auftreten erster Symptome und der finalen Diagnosestellung aufgrund Ihrer Beschwerden im Zusammenhang mit Ihrer seltenen Erkrankung in folgenden Einrichtungen des Gesundheitswesens?**

Krankenhaus (inkl. Rettungsstelle/Notaufnahme)

- ❑ 0 mal
- ❑ 1- bis 2-mal
- ❑ 3- bis 5-mal
- ❑ 6- bis 10-mal
- ❑ 11- bis 20-mal
- ❑ Mehr als 20-mal

Rettungsstelle/Notaufnahme

- ❑ 0 mal
- ❑ 1- bis 2-mal
- ❑ 3- bis 5-mal
- ❑ 6- bis 10-mal
- ❑ 11- bis 20-mal
- ❑ Mehr als 20-mal

B.5 **Welche Untersuchungen/diagnostischen Maßnahmen wurden bei Ihnen zwischen dem Auftreten erster Symptome und der finalen Diagnosestellung aufgrund Ihrer Beschwerden durchgeführt?**
(Mehrere Antworten möglich)

- ❑ Biologische Untersuchungen (z. B. Blut- oder Urintests, Lumbalpunktion, Biopsie, Knochenmarksuntersuchung, Allergietest)
- ❑ Radiologische Untersuchungen (z. B. Ultraschall, CT, MRT)
- ❑ Funktionelle Tests (z. B. Atmung, Bewegung, Koordination)
- ❑ Genetische Tests für die Diagnose der zugrunde liegenden Erkrankung
- ❑ Andere *(bitte angeben)*: ____
- ❑ Daran kann ich mich nicht erinnern
- ❑ Keine

B.6 **Wurden bei Ihnen aufgrund der Symptome der seltenen Erkrankung andere Erkrankungen festgestellt, die sich später nicht bestätigten?**

- ❑ Nein
- ❑ Ja *(bitte folgende Tabelle ausfüllen)*

Diagnose	Wann wurde diese Diagnose gestellt?	Wer hat diese Diagnose gestellt?		
		Hausarzt	Facharzt *(bitte Fachrichtung angeben)*	Kranken-haus
	Alter: ______ oder<br>Datum: ____/____ (Monat/Jahr)	❑	❑_____________	❑
	Alter: ______ oder<br>Datum: ____/____ (Monat/Jahr)	❑	❑_____________	❑
	Alter: ______ oder<br>Datum: ____/____ (Monat/Jahr)	❑	❑_____________	❑
	Alter: ______ oder<br>Datum: ____/____ (Monat/Jahr)	❑	❑_____________	❑
	Alter: ______ oder<br>Datum: ____/____ (Monat/Jahr)	❑	❑_____________	❑

B.7 **Wurde eine Behandlung dieser Diagnosen, die sich später nicht bestätigt haben, gestartet?** ***(Mehrere Antworten möglich)***

❑ Nein

❑ Ja, mit folgenden **Arzneimitteln** *(bitte angeben)*:

____

❑ Ja, mit folgender **Operation** *(bitte angeben)*:

____

❑ Ja, mit folgender **psychologischen Therapie** *(bitte angeben)*:

____

❑ Ja, mit folgenden anderen Behandlungen, z. B. Physiotherapie etc. *(bitte angeben):*

____

C Erstmalige Diagnosestellung

C.1 **Wann wurde bei Ihnen erstmals das Vorliegen einer seltenen Erkrankung in Betracht gezogen?**

Alter des Patienten: ______

oder

Datum: ___/_____ (Monat/Jahr)

C.2 **Wer äußerste erstmals den Verdacht einer seltenen Erkrankung?**

- ❑ Hausarzt
- ❑ Facharzt
- ❑ Krankenhaus
- ❑ Andere Gesundheitsberufe
- ❑ Lehrer
- ❑ Familienmitglieder
- ❑ Ich selbst
- ❑ Andere *(bitte angeben)*: ____

C.3 **Wann wurde die finale Diagnose gestellt?**

Alter des Patienten: ______

oder

Datum: ___/_____ (Monat/Jahr)

C.4 **Welcher Arzt hat schlussendlich Ihre Erkrankung erkannt, an der Sie tatsächlich leiden?**

- ❑ Arzt in spezialisiertem Zentrum
- ❑ Andere *(bitte angeben)*: ____

C.5 **Wenn Sie an das Jahr vor der finalen Diagnosestellung denken, wie würden Sie Ihren Gesundheitszustand in diesem Zeitraum beschreiben?**

- ❑ Sehr gut
- ❑ Gut
- ❑ Mittelmäßig
- ❑ Weniger gut
- ❑ Schlecht
- ❑ Keine Angabe

C.6 **Wenn Sie an das Jahr vor der finalen Diagnosestellung denken, wie zufrieden waren Sie, alles in allem, mit Ihrem Leben?**

Bitte auf der Skala von 0 („ganz und gar unzufrieden“) bis 10 („ganz und gar zufrieden“) ankreuzen!

0	1	2	3	4	5	6	7	8	9	10
❑	❑	❑	❑	❑	❑	❑	❑	❑	❑	❑

D Mitteilung der Diagnose und Therapie

D.1 **Wer teilte Ihnen das Vorliegen Ihrer seltenen Erkrankung mit?**

- ❑ Hausarzt
- ❑ Facharzt
- ❑ Spezialisiertes Zentrum
- ❑ Krankenhaus
- ❑ Anderes medizinisches Personal
- ❑ Andere *(bitte angeben)*: ______________________________

D.2 **Wie wurde Ihnen die Diagnose mitgeteilt?**

- ❑ Persönlich im Rahmen eines Gesprächstermins
- ❑ Persönlich auf anderem Weg (z. B. im Flur der Arztpraxis)
- ❑ Am Telefon
- ❑ Schriftlich
- ❑ Andere *(bitte angeben)*: ______________________________

D.3 **Wurde Ihnen ein Gespräch zur Aufklärung über den Umgang mit genetisch bedingten Erkrankungen angeboten (z. B. zur Familienplanung bzw. zur Suche nach ebenfalls betroffenen Familienmitgliedern; humangenetische Beratung)?**

- ❑ Ja
- ❑ Nein

D.4 **Wie zufrieden waren Sie mit der Art und Weise bzw. mit der Situation der Diagnosemitteilung insgesamt?**

- ❑ Sehr zufrieden
- ❑ Zufrieden
- ❑ Mittelmäßig
- ❑ Unzufrieden
- ❑ Sehr unzufrieden
- ❑ Keine Angabe

D.5 **Wie geht es Ihnen, seitdem Sie eine Therapie erhalten?**

- ☐ Sehr gut
- ☐ Gut
- ☐ Zufriedenstellend
- ☐ Weniger gut
- ☐ Schlecht
- ☐ Keine Therapie erhalten

Herzlichen Dank für Ihre Mühe bzw. Ihre Mitarbeit!
