# Supplementary material for: The patient journey of patients with Fabry disease, Gaucher disease and Mucopolysaccharidosis type II: A German-wide telephone survey
Source: PLoS One. 2020 Dec 31;15(12):e0244279. doi: 10.1371/journal.pone.0244279 (PMC7775043; doi:10.1371/journal.pone.0244279)
Supplement: S2 File — (PDF) [file pone.0244279.s002.pdf]

Survey for the VISIBL study on rare diseases

Please answer the following questions and have the questionnaire ready for the telephone survey so that we can obtain as much information as possible from you on this important topic.

Please read the questions carefully. The questions provide you with additional information on whether, for example, several answer options can be ticked off or whether you can skip individual questions and do not need to answer them. All questions are addressed to the patient himself. Should another person be involved in the survey or take over the survey, please make sure that all questions are answered from the patient's perspective.

Please do not send this questionnaire! The questionnaire is only intended to prepare you for the telephone survey.

Many thanks for your cooperation!

If you have any questions regarding the implementation of the telephone survey, please contact:

Dr. Christoph Ohlmeier
IGES Institut GmbH
Friedrichstr. 180
10117 Berlin
Tel: 030 230809-0
Mail: christoph.ohlmeier@iges.com

If you have any medical questions related to the questionnaire, please contact one of the following doctors, depending on the rare disease:

Hereditary Angioedema
Prof. Dr. Markus Magerl
Charité Universitätsmedizin Berlin
Tel: 030 450 518 318
Mail: markus.magerl@charite.de

Morbus Fabry
Dr. Jens Gaedeke
Charité Universitätsmedizin Berlin
Tel: 030 450 614137
Mail: jens.gaedeke@charite.de

Morbus Gaucher
Dr. Eugen Mengel
Villa Metabolica / Universitätsmedizin Mainz
Tel: 06131 17 5754
Mail: karl-eugen.mengel@unimedizin-mainz.de

Morbus Hunter / Mukopolysaccharidosis Type II
Dr. Jörg Reinke
Villa Metabolica / Universitätsmedizin Mainz
Tel: 06131 17 5754
Mail: joerg.reinke@unimedizin-mainz.de

A Personal Data

A.1 With whom is the telephone survey conducted?

- ❑ The person concerned himself/herself
- ❑ Minor affected person (<18 years) together with legal guardian
- ❑ Parent or legal guardian representing the person concerned
- ❑ Adult affected person together with a reference person or legal guardian

A.2 When were you born?

__ __/__ __ __ __
M M / Y Y Y Y

A.3 Your sex:

Male ❑ Female ❑

A.4 Which profession do you currently practise?

- ❑ Pupil/Student
- ❑ Tainee
- ❑ Employed
- ❑ Pensioner
- ❑ Job-seeking
- ❑ Disabled
- ❑ Other *(please specify)*:

____

A.5 What is the highest educational qualification you have?

- ❑ No school leaving certificate
- ❑ 'Haupt-' or 'Realschulabschluss' or equivalent
- ❑ Abitur
- ❑ University of Applied Sciences degree
- ❑ University degree
- ❑ Doctorate/PhD
- ❑ Other *(please specify)*:

____

A.6 **Which person was primarily involved in your contacts with doctors in connection with your rare disease?**

- ❑ Parent
- ❑ Spouse or life partner
- ❑ Other family member
- ❑ Friends/acquaintances
- ❑ I have primarily organised this myself
- ❑ Other *(please specify)*: ______________________

A.7 **What is the highest educational achievement of this person?**

- ❑ No school leaving certificate
- ❑ 'Haupt-' or 'Realschulabschluss' or equivalent
- ❑ Abitur
- ❑ University of Applied Sciences degree
- ❑ University degree
- ❑ Doctorate/PhD
- ❑ Other *(please specify)*: ______________________

A.8 **Which rare disease do you have?**

- ❑ Morbus Fabry
- ❑ Morbus Gaucher
- ❑ Morbus Hunter / Mukopolysaccharidosis Type II
- ❑ Hereditary Angioedema

A.9 **Are there other people in your family affected by this rare disease?**

❑ Yes ❑ No

A.10 **How would you currently describe your state of health?**

- ❑ Very good
- ❑ Good
- ❑ Moderate
- ❑ Not so good
- ❑ Bad
- ❑ Not specified

A.11 **How satisfied are you at present, all in all, with your life?**

Please tick on the scale from 0 ("completely dissatisfied") to 10 ("completely satisfied")!

0	1	2	3	4	5	6	7	8	9	10
❑	❑	❑	❑	❑	❑	❑	❑	❑	❑	❑

B Time from onset of first symptoms to final diagnosis

B.1 **What were the first symptoms of the rare disease that you experienced?**

Symptom	When did the symptom first appear?	How often did the symptom occur before the final diagnosis was made?			
		1- bis 5-mal	6- bis 20-mal	Mehr als 20-mal	Dauer-haftes Symp-tom
	Age:_______or<br>Date:______/____(Month/Year)	❑	❑	❑	❑
	Age:_______or<br>Date:______/____(Month/Year)	❑	❑	❑	❑
	Age:_______or<br>Date:______/____(Month/Year)	❑	❑	❑	❑
	Age:_______or<br>Date:______/____(Month/Year)	❑	❑	❑	❑
	Age:_______or<br>Date:______/____(Month/Year)	❑	❑	❑	❑

B.2 **How many different doctors have you consulted between the appearance of the first symptoms and the final diagnosis of your symptoms?**

❑ 1 to 2 doctors
❑ 3 to 5 doctors
❑ 6 to 10 doctors
❑ 11 to 20 doctors
❑ More than 20 doctors

B.3 **Between the appearance of the first symptoms and the final diagnosis of your symptoms, how often have you visited the individual doctor groups listed below?**

	0 times	1 to 2 times	3 to 5 times	6 to 10 times	11 to 20 times	More than 20 times
Family doctor / GP	❑	❑	❑	❑	❑	❑
Neurologist	❑	❑	❑	❑	❑	❑
Cardiologist	❑	❑	❑	❑	❑	❑
Nephrologist	❑	❑	❑	❑	❑	❑
Ear, nose and throat doctor	❑	❑	❑	❑	❑	❑
Orthopaedist	❑	❑	❑	❑	❑	❑
Hematologist	❑	❑	❑	❑	❑	❑
Gynaekologist	❑	❑	❑	❑	❑	❑
Pediatrist	❑	❑	❑	❑	❑	❑
Dermatologist	❑	❑	❑	❑	❑	❑
Allergologist	❑	❑	❑	❑	❑	❑
Gastroenterologist / Hepatologist	❑	❑	❑	❑	❑	❑
Ophthalmologist	❑	❑	❑	❑	❑	❑
Rheumatologist	❑	❑	❑	❑	❑	❑
Psychologist/Psychiatrist	❑	❑	❑	❑	❑	❑
Other(s)	❑	❑	❑	❑	❑	❑

B.4 **Between the appearance of the first symptoms and the final diagnosis based on your rare disease symptoms, how often have you visited the following healthcare facilities?**

**Hospital (incl. rescue centre / emergency room)**	**Rescue centre / emergency room**
❑ 0 times	❑ 0 times
❑ 1 to 2 times	❑ 1 to 2 times
❑ 3 to 5 times	❑ 3 to 5 times
❑ 6 to 10 times	❑ 6 to 10 times
❑ 11 to 20 times	❑ 11 to 20 times
❑ More than 20 times	❑ More than 20 times

B.5 **What examinations/diagnostic measures have been carried out on you between the appearance of the first symptoms and the final diagnosis based on your symptoms?**
(Multiple answers possible)

❑ Biological examinations (e.g. blood or urine tests, lumbar puncture, biopsy, bone marrow examination, allergy test)

❑ Radiological examinations (e.g. ultrasound, CT, MRT)

❑ Functional tests (e.g. breathing, movement, coordination)

❑ Genetic tests for the diagnosis of the underlying disease

❑ Others *(please specify)*: ______________________________________

❑ I cannot remember this

❑ None

B.6 **Have you been diagnosed with other diseases due to the symptoms of the rare disease which were not confirmed later?**

❑ No

❑ Yes *(please fill in the following table)*

Diagnosis	When was this diagnosis made?	Who made this diagnosis?		
		Family doctor / GP	Specialist *(please specify)*	Hospital
	Age:_______or<br>Date:______/____(Month/Year)	❑	❑___________	❑
	Age:_______or<br>Date:______/____(Month/Year)	❑	❑___________	❑
	Age:_______or<br>Date:______/____(Month/Year)	❑	❑___________	❑
	Age:_______or<br>Date:______/____(Month/Year)	❑	❑___________	❑
	Age:_______or<br>Date:______/____(Month/Year)	❑	❑___________	❑

B.7 **Has treatment been started for these diagnoses, which have not been confirmed?**
(Multiple answers possible)

❑ No

❑ Yes, with the following d**rugs** *(please specify)*:

____

❑ Yes, with the following **surgical intervention** *(please specify)*:

____

❑ Yes, with the following **psychological therapy** *(please specify)*:

____

❑ Yes, with the following other treatments, e.g. physiotherapy etc. *(please specify):*

____

C Initial Diagnosis

C.1 When did you first consider the presence of a rare disease?

Age of the patient:________

or

Date:_____/_____ (Month/Year)

C.2 Who first suspected a rare disease?

- ❑ Family doctor / GP
- ❑ Specialist
- ❑ Hospital
- ❑ Other health professionals
- ❑ Teacher
- ❑ Family member
- ❑ I myself
- ❑ Others *(please specify)*: ____

C.3 When was the final diagnosis made?

Age of the patient:________

or

Date:_____/_____ (Month/Year)

C.4 Which doctor finally identified the disease you actually suffer from?

- ❑ Doctor in specialised centre
- ❑ Others *(please specify)*: ____

C.5 If you think back to the year before the final diagnosis, how would you describe your health status during this period?

- ❑ Very good
- ❑ Good
- ❑ Moderate
- ❑ Not so good
- ❑ Bad
- ❑ Not specified

C.6 **When you think back to the year before the final diagnosis, how satisfied you were, all in all, with your life?**

Please tick on the scale from 0 ("completely dissatisfied") to 10 ("completely satisfied")!

0	1	2	3	4	5	6	7	8	9	10
❑	❑	❑	❑	❑	❑	❑	❑	❑	❑	❑

D Communication of Diagnosis and Therapy

D.1 **Who informed you of the presence of your rare disease?**

- ❑ Family doctor / GP
- ❑ Specialist
- ❑ Specialized Centre
- ❑ Hospital
- ❑ Other medical personnel
- ❑ Others *(please specify)*: ____

D.2 **How were you informed of the diagnosis?**

- ❑ In person at a meeting/consultation
- ❑ Personally by other means (e.g. in the corridor of the doctor's office)
- ❑ By phone
- ❑ In writing
- ❑ Others *(please specify)*: ____

D.3 **Have you been offered an interview to explain how to deal with genetic diseases (e.g. for family planning or to find family members who are also affected; genetic counselling)?**

- ❑ Yes
- ❑ No

D.4 **How satisfied were you with the way in which the diagnosis was communicated and with the situation as a whole?**

- ❑ Very satisfied
- ❑ Satisfied
- ❑ Moderate
- ❑ Unsatisfied
- ❑ Very unsatisfied
- ❑ Not specified

D.5 **How do you feel since you have received therapy?**

- ❑ Very good
- ❑ Good
- ❑ Moderate
- ❑ Not so good
- ❑ Bad
- ❑ No therapy received

Many thanks for your effort and cooperation!
